# Polyunsaturated fatty acids modify expression of TGF‐β in a co‐culture model ultilising human colorectal cells and human peripheral blood mononuclear cells exposed to *Lactobacillus gasseri*, *Escherichia coli* and *Staphylococcus aureus*

**DOI:** 10.1002/ejlt.201300337

**Published:** 2014-03-31

**Authors:** Kerry L Bentley‐Hewitt, Cloe Erika De Guzman, Juliet Ansell, Tafadzwa Mandimika, Arjan Narbad, Elizabeth K Lund

**Affiliations:** ^1^ Institute of Food ResearchNorwichNorfolkUK; ^2^ Food and NutritionThe New Zealand Institute for Plant & Food Research LimitedPalmerston NorthNew Zealand

**Keywords:** EPA, Fish oil, Lactobacilli, TGF‐β

## Abstract

Commensal bacteria and polyunsaturated fatty acids (PUFAs) have both been shown independently to modulate immune responses. This study tested the hypothesis that the different colonic immunomodulatory responses to commensal (*Lactobacillus gasseri*) and pathogenic bacteria (*Escherichia coli* and *Staphylococcus aureus*) may be modified by PUFAs. Experiments used a Transwell system combining the colorectal cell line HT29, or its mucous secreting sub‐clone HT29‐MTX, with peripheral blood mononuclear cells to analyse immunomodulatory signalling in response to bacteria, with and without prior treatment with arachidonic acid, eicosapentaenoic acid or docosahexaenoic acid. *L. gasseri* increased transforming growth factor β1 (TGF‐β1) mRNA and protein secretion in colonic cell lines when compared with controls, an effect that was enhanced by pre‐treatment with eicosapentaenoic acid. In contrast, the Gram‐negative pathogen *E. coli* LF82 had no significant effect on TGF‐β1 protein. *L. gasseri* also increased IL‐8 mRNA but not protein while *E. coli* increased both; although differences between PUFA treatments were detected, none were significantly different to controls. Colonic epithelial cells show different immunomodulatory signalling patterns in response to the commensal *L. gasseri* compared to *E. coli* and *S. aureus* and pre‐treatment of these cells with PUFAs can modify responses.

**Practical applications:** We have demonstrated an interaction between dietary PUFAs and epithelial cell response to both commensal and pathogenic bacteria found in the gastrointestinal tract by utilising in vitro co‐culture models. The data suggest that n‐3 PUFAs may provide some protection against the potentially damaging effects of pathogens. Furthermore, the beneficial effects of combining n‐3 PUFAs and the commensal bacteria, and potential probiotic, *L. gasseri* are illustrated by the increased expression of immunoregulatory TGF‐β1.

**Abbreviations**
AA: arachidonic acid
ACTB: beta actin
BHI: brain heart infusion broth
DHA: docosahexaenoic acid
EPA: eicosapentaenoic acid
GAPDH: glyceraldehyde 3‐phosphate dehydrogenase
HSP: heat shock protein
IL‐8: interleukin‐8
MRS: Mann, Rogosa and Sharpe media
PBMC: peripheral blood mononuclear cell
PPAR: proliferator‐activated receptor
PUFA: polyunsaturated fatty acid
RT‐qPCR: reverse‐transcription qPCR
TGF‐β1: transforming growth factor beta
TLR: toll‐like receptors
TNF‐α: tumour necrosis factor alpha
TSB: trypticase soy broth

## 1 Introduction

It is now well recognised that probiotic bacteria, such as lactobacilli, can modulate immune responses and improve conditions such as inflammatory bowel disease 1. There is also evidence that PUFA may modify the risk and impact of inflammatory bowel disease through their immunomodulatory effects 2. Functional foods may have one or both of these potentially advantageous factors added to enhance health benefits. Furthermore, there is good evidence of a symbiotic effect between dietary PUFAs and probiotic bacteria in which n‐3 PUFAs have been shown to increase adhesion of beneficial lactobacilli in the intestines of piglets and Arctic Charr 5. Both pro‐ and anti‐inflammatory effects of lactobacilli 7 and PUFAs 11 have previously been reported. These studies suggest that these food components function in an immunomodulatory homeostatic manner rather than completely inhibiting or stimulating a particular type of inflammatory response. In a healthy individual, a compromise between pro‐ and anti‐inflammatory responses is needed to maintain appropriate immune surveillance to protect the host from invading pathogens and allow the survival of beneficial commensal bacteria through the avoidance of an excessive immunological response. Therefore, colonic epithelial cells must distinguish between potential threats from pathogenic bacteria and benefits from resident commensals.

Epithelial responses to luminal bacteria are known to be mediated through a number of pattern recognition molecules such as toll‐like receptors (TLRs) that allow recognition of specific bacterial antigens. TLR 2 recognises peptidoglycan present in gram‐positive bacteria, such as *Lactobacillus* species 14. TLR 2 activation leads to transcription of NF‐κB regulated genes including cytokines and chemokines, such as IL‐8, that aid in the clearance of pathogenic bacteria by attracting immune cells to the site of activation 15. However, TLRs have also been implicated in mediating control of tissue homeostasis. For example, TLR 9, which responds to immune‐stimulatory bacterial CpG‐DNA, has been reported to mediate the anti‐inflammatory effects of probiotics 16. Additionally, they can up‐regulate heat shock proteins (HSPs) that are well established to play a cytoprotective role in intestinal epithelial cells 17.

The immunomodulatory effects of PUFAs may arise as a result of a number of potential mechanisms 3. It is known that PUFAs are effectively incorporated into colorectal cell lines in culture 18. Here they can increase membrane fluidity and alter the function and activity of cell surface proteins 19. Alternatively they may modify gene expression; they and their eicosanoid and docosanoid metabolites are recognised ligands for PPAR. For example, docosahexaenoic acid (DHA) binding to PPAR γ can lead to activation, which in turn has been reported to increase expression of anti‐inflammatory cytokines, such as IL‐10 in dendritic cells, that can dampen the pro‐inflammatory response 21. Of particular relevance to the present study are the reports that PPAR δ activation up‐regulates TGF‐β 22. TGF‐β is involved in oral tolerance of bacteria, and therefore aids in bacteria survival 23.

To observe immunomodulation by dietary factors using a colonic model system, it is necessary to have components of the immune system present, such as the white blood cells, since intestinal epithelial cells can regulate innate immune responses by directly interacting with dendritic cells, lamina propria lymphocytes and intra‐epithelial lymphocytes 26. To achieve this, Haller et al. 26 have previously employed a co‐culture Transwell system with intestinal epithelial cell lines to represent the gut epithelium in the top compartment and peripheral blood mononuclear cells (PBMC) to represent immune cells present in the lamina propria in the bottom compartment of the Transwell. This system allows cross talk between the two compartments, theoretically reflecting that between the gut epithelium and associated immune cells.

Since both PUFAs and lactobacilli have strong immunomodulatory effects the aim of this study was to test whether PUFAs could modify epithelial cell immune responses to lactobacilli and potentially identify a common pathway by which these two components might play a role in physiological immune homeostasis. To allow the comparison with a more pathological response, we also tested two potentially pathogenic microbial strains (gram‐positive and gram‐negative) in the Transwell system. The changes in immunomodulatory signalling were measured in HT29 and mucous secreting HT29‐MTX cells 27 exposed to either lactobacilli or pathogenic bacteria in combination with different PUFAs, to ensure results were replicable in another cell line and to investigate the role of mucus. Furthermore, the possibility that difference in gene expression might be linked to differences in PPAR protein production in different cell lines and in response to different PUFAs was investigated.

## 2 Materials and methods

### 2.1 Study design

Two sets of experiments were performed. First, the effects of the probiotic bacteria *L. gasseri* on gene and protein production were examined in two colorectal cell lines, HT29 and the mucous secreting HT29‐MTX cells, with and without pre‐incubation with different PUFAs. In the second set we assessed whether similar effects were seen in response to two pathogenic strains.

### 2.2 Culture of human cell lines with PUFA and PBMC

Human intestinal cell lines; HT29 (European Tissue Culture Collection, Dorset, UK) and HT29‐MTX (Thécla Lesuffleur, Paris) were grown in Roswell Park Memorial Institute medium (RPMI) + Glutamax (10% v/v foetal calf serum, 2% v/v penicillin–streptomycin) from the frozen stock between 2 and 10 passages. Epithelial cells were grown in six‐well polyester transwell plates (0.4 µm pore size, inserts of 24 mm diameter) (Corning, New York, USA), seeded at 50 × 10^4^ cells/mL until confluent, then washed in RPMI + Glutamax three times. Aliquots of 50 µM eicosapentaenoic acid (EPA), DHA, arachidonic acid (AA) all reconstituted in ethanol or equivalent ethanol control were added in RPMI + Glutamax (5% foetal calf serum) to apical side of cell lines. This concentration of PUFA has been previously shown in our labs to give optimal uptake of PUFA into the cell lines without causing toxicity. Additionally, based on a study showing that 2% of dietary fatty acids may escape absorption in the small intestine 28 a 50 µM concentration physiologically relevant to a diet containing foods rich in fish oils. Peripheral blood mononuclear cells (PBMCs) were isolated from 200 mL blood (New Zealand Blood Service) pooled from four healthy adult individuals, using 1.077 g/mL Histopaque (Sigma) following manufacturer instructions, and suspended in RPMI + Glutamax (5% v/v foetal calf serum). PBMCs (1.5 mL of 1.9 × 10^6^ cells/mL) were added to the basolateral side of cells at the same time as PUFAs were added to the apical side and the co‐culture was incubated for 12 h. The apical side of cells were then washed three times in RPMI + Glutamax without foetal calf serum or antibiotics prior to incubation with bacteria.

### 2.3 Bacterial culture and co‐incubation of bacteria with human cell lines

Human isolates *Lactobacillus gasseri* ATCC 33323, *Escherichia coli* LF82 and *Staphylococcus aureus* ATCC 25923 were grown in De‐mann, Rogosa and Sharpe media (MRS), trypticase soy broth (TSB) or brain heart infusion broth (BHI), respectively. *L. gasseri* was inoculated at 1% in MRS media and *E. coli* and *S. aureus* at 0.1% into fresh TSB or BHI broth, respectively and grown overnight at 37°C. Bacteria were then washed three times in phosphate buffered saline (PBS) and re‐suspended in 1 mL RPMI + Glutamax. *L. gasseri* (1 × 10^9^), *E. coli* LF82(1 × 10^6^) and *S. aureus* (1 × 10^6^) in RPMI + Glutamax or RPMI alone (control). The bacteria were then added to the apical side of HT29 and HT29‐MTX cells (previously treated with PUFA and PBMC) and incubated for 3 h at 37°C and 5% CO_2_. Numbers of bacteria were lowered for pathogenic species since preliminary experiments had shown that cell lines were damaged following exposure to higher cell numbers: whilst 10^9^ lactobacilli are representative of numbers found in the colon, pathogenic bacteria inflict substantial infection at lower levels. Each treatment was represented by three biological replicates (wells) in a tissue culture plate. Non‐adherent bacteria were then removed by washing in RPMI three times. Fresh RPMI + Glutamax was then added to the apical surface for a further 6 h to allow for optimal protein production after which apical supernatants were collected for protein analysis by ELISA, prior to cells removal by applying 300 µL RNA extraction lysis buffer (QIAGEN—RNeasy Mini Kit) directly onto the transwell insert membrane. Samples were then stored at −80°C.

### 2.4 Gene expression analysis

RNA was extracted using the RNeasy Mini Kit (Qiagen, Doncaster, Australia) in combination with the RNase‐free DNase kit (Qiagen) carried out according to the manufacturer's instructions. RNA yield and purity (1.8 < OD_260_/OD_280_ < 2.0) was measured using a NanoDrop® ND‐1000 (NanoDrop Technologies, Inc., Wilmington, DE, USA) while RNA integrity was determined by agarose gel electrophoresis. All reverse transcription reactions were done using 1 µg of total RNA with the Transcriptor First Strand cDNA Synthesis kit (Roche, Auckland, New Zealand) according to manufacturer's instructions for oligo‐dT primed reactions. The cDNA was stored at −20°C. Primers and primer‐probe combinations (Table 1) were designed online using the Universal probe library system assay design centre (Roche Applied Science). After primer design, all primers were run through the National Center for Biotechnology Information (NCBI) Blast database to check for specificity. Dual‐hybridisation probes from the Universal Probe Library (Roche Diagnostics, Manheim, Germany) were paired with unmodified, desalted primers (Invitrogen New Zealand Ltd.). A manually set‐up, 96‐well format, reverse‐transcription qPCR (RT‐qPCR) assay was performed using the Lightcycler® 480 system (Roche Diagnostics, Manheim, Germany) with three reactions (technical replicates) for each sample. Each reaction contained 5 µL of cDNA template, primers (200 mmol/L), probes (100 mmol/L), and Lightcycler® 480 Probes Master (FastStart Taq DNA Polymerase, 6.4 mmol/L MgCl_2_; Roche). Real‐time PCR parameters were as follows: 10 min (0:10:00) pre‐incubation at 95°C, 40 cycles of amplification from 95°C (0:00:10), to 58°C (0:00:20), to 72°C (0:00:01), followed by cooling at 40°C (0:00:10). No‐template‐controls included in reverse‐transcription reactions and RT‐qPCR runs were negative for amplification products. Standard curves for each gene and cell line were generated on separate runs using up to seven serial dilutions (1/10–1/1000) of pooled cDNA samples.

**Table 1:** qPCR oligonucleotides and RT‐qPCR efficiencies for the cells/cell lines (i) HT29 AND (ii) HT29‐MTX

Gene name	Genebank access no.	Primer and probe sequences	Amplicon size
GAPDH (glyceraldehyde‐3‐phosphate dehydrogenase)	NM_002046.3	F: AGCCACATCGCTCAGACAC	66
		R: GCCCAATACGACCAAATCC	
		Probe #60: TGGGAAG	
HSP 72 (heat shock 70 kDa protein 1a)	NM_005345.4	F: GGAGTCCTACGCCTTCAACA	89
		R: CCAGCACCTTCTTCTTGTCG	
		Probe #88: GGAGGATG	
IL‐8 (interleukin 8)	NM_000584.2	F: AGACAGCAGAGCACACAAGC	62
		R: ATGGTTCCTTCCGGTGGT	
		Probe #72: GCCAGGAA	64
TGF‐β1 (transforming growth factor β1)	NM_000660.3	F: GCAGCACGTGGAGCTGTA	
		R: CAGCCGGTTGCTGAGGTA	
		Probe #72: TTCCTGGC	

A preliminary assessment of candidate gene expression in the cell lines under investigation following exposure to PBMC was made in pooled cDNA samples before analysis of individual samples. The genes selected for analysis were housekeeping genes glyceraldehyde 3‐phosphate dehydrogenase (GAPDH) and beta actin (ACTB), also HSP 25, HSP 72, transforming growth factor β (TGF‐β1), interleukin‐8 (IL‐8), tumour necrosis factor alpha (TNF‐α), interleukin‐10, interleukin‐2, nucleotide‐binding oligomerisation domain 2, TLR‐4, interferon γ and GATA binding protein 3 selected based on their previous association with responses to PUFAs and commensals 29. Genes were selected for further study from those detectable by RT‐PCR within 30 cycles. The final genes analysed were IL‐8, TNF‐α, TGF‐β1, HSP 25 and HSP 72, with reference gene GAPDH (ACTB did not produce a usable standard curve in 30 cycles and was omitted). We have not included methods or results for TNF‐α, and HSP 25 expression as no significant effects of treatments (bacteria or PUFA) were detected.

### 2.5 Protein production analysis

IL‐8 and TGF‐β1 protein production were analysed according to the manufacturer's instructions using ELISA kits from Invitrogen (Catalog No. KHC0081 and KAC1688, respectively). The effects of PUFAs were only analysed for TGF‐β1 as PUFAs had no effect on IL‐8 gene expression relative to controls. HSP 72 was not analysed as the commensal *L. gasseri* had only a minimal effect on gene expression.

### 2.6 Peroxisome proliferator‐activated receptor (PPAR) *Transcription Factor Assay*

HT29 and HT29‐MTX cells were grown in 75 cm^2^ cell culture flasks as described previously until confluent. Cell were treated for 48 h with either 50 µM AA, EPA, DHA or equivalent ethanol (control) in duplicate and removed with 6 mL 0.05% v/v Trypsin 0.53 mmol/L 53 mM EDTA.4Na (Invitrogen). Nuclear extracts were isolated using a “Nuclear Extraction Kit” (Cayman Chemical, Catalog No. 10009277) and samples were tested for protein concentration using BCA Protein Assay Kit (Pierce). Nuclear extracts were added to the PPARα, δ, γ ‘Complete Transcription Factor’ Assay plate (Cayman Chemical, Catalog No. 10008878) using each biological replicate and testing for each PPAR following manufacturer's instructions. Sample optical density (450 nm) was corrected for protein concentration.

### 2.7 Statistical analysis

Quantitative RT‐PCR results were analysed using inbuilt relative quantification software (Light–Cycler 480 software version 1.0), using the standard curve for both target and reference (GAPDH) gene, where the software determined the target to reference ratio.

Triplicate values for each immunomodulatory molecule (mRNA or protein) were normalised to controls to make more accurate comparisons between commensal and pathogen experiments. The resulting data were analysed by two‐way ANOVA (PUFA vs. bacterial treatments) and one‐way ANOVA to analysis PUFA effect in individual bacteria treatment groups, using the General Linear Model with Tukey comparisons (Minitab version 15). Differences with *p* < 0.05 were considered statistically different.

## 3 Results

### 3.1 TGF‐β1, IL‐8 and HSP 72 gene expression in response to *L. gasseri*, *E. coli* LF82 and *S. aureus*

**Figure 1:**
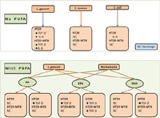
Gene expression (HSP 72, IL‐8 and TGF‐β1) in HT29 and HT29‐MTX cells co‐cultured with PBMC following exposure to bacteria. Gene expression (HSP 72, IL‐8 and TGF‐β1) following exposure of (a) HT29 and (b) HT29‐MTX cells to *L. gasseri*, *E. coli* LF82 and *S. aureus*. Individual results were normalised to GAPDH and expressed relative to experimental controls where cells are not exposed to bacteria. Each bar represents mean ± SD (*n* = 3). Significant differences relative to no bacteria with no PUFAs added are shown as *(*p* < 0.05).

*L. gasseri* exposure resulted in an increase in TGF‐β1 mRNA compared with the control (no bacteria) in HT29 and HT29‐MTX cells. In contrast, expression of TGF‐β1 mRNA was unchanged in the pathogen exposed cells. *L. gasseri* exposure increased IL‐8 gene expression by 160‐ and 10‐fold in both HT29 and HT29‐MTX cells, respectively, while the Gram‐negative pathogen *E. coli* LF82 increased IL‐8 expression in HT29‐MTX cells only. No significant difference in gene expression was seen following *S. aureus* exposure to either cell line (Fig. 1).

### 3.2 Polyunsaturated fatty acids: AA, EPA and DHA modify TGF‐β1, HSP 72 and IL‐8 gene expression

**Figure 2:**
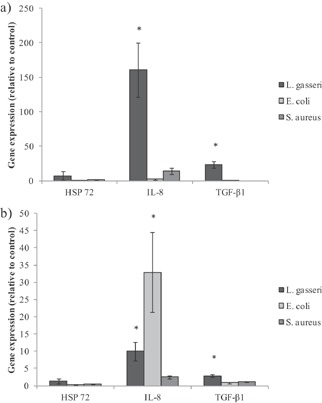
TGF‐β1 gene expression in HT29 and HT29‐MTX cells co‐cultured with PBMC following PUFA treatment and exposure to *L. gasseri*. Gene expression (TGF‐β1) following PUFA treatment of (a) HT29 and (b) HT29‐MTX exposed to *L. gasseri* or no bacteria. Individual results were normalised to GAPDH and expressed relative to no PUFA controls to compare PUFA effects in the absence of bacterial exposure. Each bar represents the mean ± SD (*n* = 3). Significant differences relative to the control are shown as *(*p* < 0.05).

TGF‐β1 gene expression in HT29 cells not exposed to any bacteria was increased fourfold by all tested PUFAs (Fig. 2a), but this effect was completely annulled following *L. gasseri* exposure, except in EPA pre‐treated cells where a small increase above control levels was observed. In HT29‐MTX cells only, EPA significantly increased TGF‐β1 gene expression in the absence of bacteria and *L. gasseri* treatment counteracted this effect, while in AA and DHA pre‐treated cells expression dropped below control values, albeit not significantly (Fig. 2). No significant differences in TGF‐β1 expression between PUFA treatments were found following pathogen exposure (data not shown).

IL‐8 and HSP 72 gene expression was not consistently modified by PUFA following bacterial exposure in either cell line. However, results indicate a decrease in HSP 72 expression following pre‐treatment with PUFA and *S. aureus* exposure (Table 2).

**Table 2:** HSP 72 and IL‐8 gene expression in HT29 and HT29‐MTX cells following PUFA treatment and bacterial exposure

Cell line	Fatty acid	*L. gasseri*				*E. coli LF82*				*S. aureus*			
		HSP72		IL‐8		HSP72		IL‐8		HSP72		IL‐8	
		Mean	SD	Mean	SD	Mean	SD	Mean	SD	Mean	SD	Mean	SD
HT29	AA	5.37	3.12	1.12	0.22	0.57	0.40	1.13	0.22	0.37	0.07*	1.65	0.81
	EPA	1.38	1.01	1.39	0.22	3.10	0.56*	3.36	3.56	0.74	0.09*	0.31	0.13
	DHA	3.65	1.72	0.83	0.04	1.69	0.64	7.16	2.92*	0.51	0.05*	0.62	0.25
HT29‐MTX	AA	0.65	0.30	0.31	0.24	1.18	0.12	1.45	0.22	1.04	0.04	1.19	0.50
	EPA	0.82	0.42	0.97	0.51	1.15	0.06	1.01	0.17	1.11	0.22	1.77	0.42
	DHA	2.79	2.13	0.64	0.25	1.12	0.17	0.80	0.14	1.24	0.20	1.29	0.56

Gene expression (HSP 72 and IL‐8) normalised to GAPDH following PUFA treatment and bacterial exposure expressed relative to control treatment within each bacterial exposure to exclude bacterial effect. Each result represents mean ± SD, *n* = 3. Significant differences between PUFA treatment and control are indicated by * (*p* < 0.05).

### 3.3 TGF‐β1 and IL‐8 protein production in response to *L. gasseri*, *E. coli* LF82 and *S. aureus*

**Figure 3:**
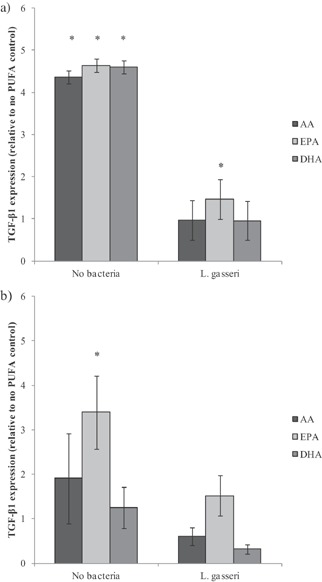
TGF‐β1 protein production from HT29 and HT29‐MTX cells co‐cultured with PBMC and exposed to bacteria. TGF‐β1 protein production (pg/mL) following exposure of (a) HT29 (b) HT29‐MTX cells to bacteria. Results are normalised to experimental controls where cells were not exposed to bacteria. Each bar represents the mean ± SD (*n* = 3). Significant differences between bacterial exposure and the ‘no bacteria’ control are shown as *(*p* < 0.05).

As with gene expression analysis, TGF‐β1 protein was increased in response to *L. gasseri* exposure in both cell lines, whilst exposure to *E. coli* LF82 had no effect on TGF‐β1 protein production in either cell line and *S. aureus* increased production in HT29 cells only (Fig. 3). Differences in TGF‐β1 protein production between PUFA pre‐treatment and control were tested in *L. gasseri* exposed cells; although no significant differences were found, similar patterns to those for gene expression following PUFA pre‐treatment were observed. For example, EPA increased TGF‐β1 protein by 23 and 21% in HT29 and HT29‐MTX cells, respectively (data not shown).

**Figure 4:**
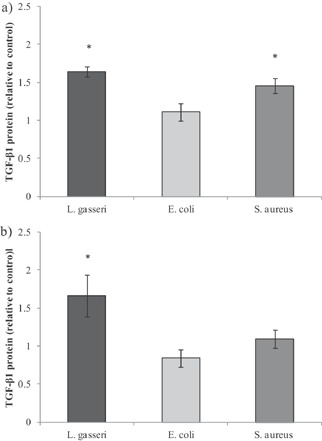
IL‐8 protein production from HT29‐MTX cells co‐cultured with PBMC and exposed to bacteria. IL‐8 protein production (pg/mL) following exposure of HT29‐MTX cells to bacteria. Results are normalised to experimental controls where cells were not exposed to bacteria. Each bar represents the mean ± SD (*n* = 3). Significant differences between bacterial exposure and the ‘no bacteria’ control are shown as *(*p* < 0.05).

*L. gasseri* exposure led to a threefold increase in IL‐8 (1.11 ± 0.02 ng/mL) compared with control HT29 cells (0.38 ± 0.09 ng/mL) but no detectable protein was found in *E. coli* LF82 and *S. aureus* co‐incubations in HT29 cells (data not shown). However, in the HT29‐MTX cells/cell line there was an increase of IL‐8 protein following Gram‐negative *E. coli* LF82 exposure, whilst there was no change after Gram‐positive *L. gasseri* or *S. aureus* exposure (Fig. 4).

### 3.4 PPAR production following AA, EPA and DHA treatment

HT29‐MTX cells expressed high levels of PPAR δ, and PPAR γ compared with HT29 cells (relative ratio 3.08, 2.94, respectively). PUFA treatment had no effect on PPAR concentration in either HT29 cells and in HT29‐MTX (data not shown).

## 4 Discussion

The gut surface is constantly exposed to commensal bacteria that elicit no adverse immune responses in healthy, tolerant individuals while appropriate responses to pathogens can still be induced. Thus, a complex signalling environment exists at the mucosa to achieve an appropriate response. In this study we have investigated how different PUFAs might affect immunological responses to both commensals and pathogens *in vitro*. A single cell model system cannot effectively represent the complexity of the human gastrointestinal tract, therefore to obtain more physiologically relevant results, two cell lines isolated from the human colon were used in a co‐culture system with PBMC. This model, developed from that described by Haller et al. 26 allows for cell‐to‐cell cross talk that is an important part of many cytokine responses. The data we report here reflect a complex interaction between bacteria, colonocyte cell line and different dietary PUFA in relation to epithelial cell gene expression of TGF‐β1, IL‐8 and HSP 72.

The impact of PUFAs on cytokine responses was most interesting in respect to TGF‐β1 where EPA increased both gene and protein expression in the two cell lines, although the protein did not reach significance. In HT29 cells all PUFAs tested increased expression, in the absence of bacteria, to a similar extent but exposure to *L. gasseri* caused much greater increases and these were further augmented by pre‐treatment with EPA. In contrast, *L. gasseri* had relatively little effect on the more differentiated HT29‐MTX sub‐clone: here the specific effect of EPA was apparent both in the presence and absence of *L. gasseri*. Experiments in our labs have shown that the percentage of total cellular and phospholipid fraction EPA and DHA increase in both HT29 and HT29‐MTX cells, whilst the percentage of AA only increased in HT29‐MTX cells. Interestingly, the percentage of EPA in the phospholipid fraction of cells treated with EPA was significantly higher that the percentage of AA and DHA following their respective treatments. This may explain why EPA is more effective in TGF‐β up‐regulation compared to the other PUFAs tested in this study. The difference in response between the two cell lines to the gram‐positive bacteria *L. gasseri* may be related to differences in expression of pattern recognition receptors 36 or bacterial adhesion 38. The literature directly comparing the two cell lines is somewhat limited. It has been reported that these cell lines do not express TLR 2; however, TLR 4 (which recognises gram‐negative lipopolysaccharide) is expressed in HT29 cells but not in the differentiated HT29‐MTX line 39. Therefore the down regulation of TGF‐β1 gene expression in response to *E. coli*, which was seen only in HT29 cells in the current study, may be associated with increased TLR 4 protein production. Interestingly our results using HT29 cell are supported by ex vivo experiments 40, whilst previous studies using HT29 cells in a single cell system have not reported this effect 41, supporting the concept that leukocytes play an important role in epithelial immune signalling. TGF‐β is known to be important in regulating colonic inflammation 42 and therefore the increase in expression in cells treated with the probiotic *L. gasseri* or the n‐3 PUFA EPA is consistent with their reported anti‐inflammatory properties. Furthermore, TGF‐β1 was not up regulated by pathogenic *E. coli*. TGF‐β has been associated with oral tolerance towards commensal bacteria 17. The increase in TGF‐β following exposure to probiotic *L. gasseri* but not to pathogenic *E. coli* may indicate a mechanism in which *L. gasseri* can promote its own survival in the human gut. It is interesting to note that production of TGF‐β protein was much higher in the more differentiated HT29‐MTX cell line, perhaps suggesting production had been maximised and could not be further enhanced by the presence of *L. gasseri*. However, a further enhancing effect of EPA was detectable both in the presence and absence of *L. gasseri* in the HT29‐MTX cells, an effect which may be linked to the higher concentration of PPARs found in this cell line, as PUFAs and their oxidative metabolites are recognised ligands for the nuclear receptors which are known to modulate inflammation in colonocytes 44. Additionally, TGF‐β expression has previously been shown to be a target for PPAR δ 22.

Our results confirm previous studies showing that epithelial cells not only respond to the presence of pathogens but also to commensals by increasing gene expression of the chemokine IL‐8 41. However, in contrast to effects on TGF‐β1, PUFAs had no consistent effect on IL‐8 expression. A direct comparison between the effects of pathogens and commensals cannot be made as the results are based on independent experiments, but we did observe that expression of IL‐8 is significantly increased in both HT29 cells and the mucous secreting HT29‐MTX sub‐clone in response to *L. gasseri*. In contrast, the two cell lines responded differently to the two pathogens such that *S. aureus* increased expression in HT29 cells and *E. coli* increased expression in the MTX sub‐clone. This would suggest that the presence of mucins modifies bacterial signalling; however, we cannot ignore the possibility that this may also be due to differences in receptor expression and subsequent signalling. For example, the higher levels of TLR 4 and CD14 expressed in the parental HT29 cells as compared with the sub‐clone 39 might predict a stronger IL‐8 response to *E. coli* in the parent line. However, we observed a greater IL‐8 response to *E. coli* in the MTX clone at both the gene and protein level. This difference may relate to the presence of PBMC in our experiments but this was not investigated within this study. A similar lack of responsiveness following HT29 cell exposure to *E. coli* has been reported previously 30. Our result may indicate a potential mechanism by which Gram‐positive *L. gasseri* and *S. aureus* may increase IL‐8 expression in this cell line and the failure for this to be translated into increased protein production is presumably due to post‐transcriptional regulation.

## 5 Conclusions

We have demonstrated an interaction between dietary PUFAs and epithelial cell response to both commensal and pathogenic bacteria found in the gastrointestinal tract by utilising in vitro co‐culture models. This prelimary data suggests that n‐3 PUFAs may provide some protection against the potentially damaging effects of pathogens. Furthermore, the beneficial effects of combining n‐3 PUFAs and the commensal bacteria, and potential probiotic, *L. gasseri* are illustrated by the increased expression of immunoregulatory TGF‐β1.

*This work was funded by a Biotechnology and Biological Science Research Council doctoral training grant award to the Institute of Food Research and also the Ministry of Business, Innovation and Employment (formerly Foundation for Research Science and Technology New Zealand). We thank David Lewis for use of his extensive laboratory facilities and New Zealand Blood Service for the blood supplied for experiments*.

*The authors have declared no conflict of interest*.

***Author contributions*:** *K. L. Bentley‐Hewitt, E. K. Lund, T. Mandimika and J. Ansell designed the research. K. L. Bentley‐Hewitt, E. De Guzman conducted the research and performed statistical analysis of data. K. L. Bentley‐Hewitt and E. K. Lund wrote the paper and A. Narbad provided expertise in commensal microbiology. E. K. Lund has primary responsibility for final content*.
